# Problems in the management of mass casualties in the Tianjin explosion

**DOI:** 10.1186/s13054-016-1224-6

**Published:** 2016-02-26

**Authors:** Hong-Yu Wang, Hai Yan Wu

**Affiliations:** PLA No. 254 Hospital, Affiliated Hospital of Medical College of Nankai University, HuangWei Road, Tianjin, 300000 China; PLA No. 302 Hospital, West Fourth Ring Road, Beijing, 100000 China

Not long ago, a catastrophic explosion occurred in Tianjin, generating mass casualties. Recent data suggest that the number of victims was nearly 200, which included 99 firefighters. Our hospital took part in the emergency rescue and later provided treatment to critically ill patients. We have reviewed the problems that we encountered there in the management of mass casualties.

Firstly, many doctors and on-the-scene rescuers lacked the concept of triage or the training to carry it out. After the explosion, on-site doctors first provided treatment to the patients they encountered initially. The doctors should have classified the mass casualties and provided treatment to patients who most needed the first aid [1, 2]. Opening the airway in a timely fashion, stopping active bleeding, and performing cardiopulmonary resuscitation could have saved more lives in this situation.

Secondly, aid agencies and the hospitals lacked an awareness of classification and delivery. Doctors always tried to cure these patients completely and rarely transported the injured to a rear hospital. So lots of victims were turned away for lack of space to treat them. The front rescue hospitals should have played the role of the primary treatment institutions and transferred the patients with stable vital signs who had received preliminary treatment [3].

Thirdly, we should pay more attention to the importance of damage control surgery in the treatment of mass casualties [4, 5]. Some of the front hospitals spent too much time and energy implementing sophisticated operations such as vascular anastomosis.

Not only did this mean that patients who needed surgery were unable to undergo surgery, but the outcome of the operations was often poor, resulting in the need for further procedures at a later date. In addition, health authorities and aid workers had not checked the wounded (fireman and military rescuers) to see whether they were carrying concealed weapons, explosives, or other dangerous goods. Because they may be suffering from traumatic stress disorder, patients carrying such items will also be dangerous.

The management of mass casualties after a sudden accident is different from the conventional provision of treatment to patients in a hospital. A better understanding of concepts and principles in the management of mass casualties will greatly improve the rate of success. We should draw lessons from the blood and train the medical staff and rescue workers to learn the relevant knowledge.
